# Bayesian approaches to reverse engineer cellular systems: a simulation study on nonlinear Gaussian networks

**DOI:** 10.1186/1471-2105-8-S5-S2

**Published:** 2007-05-24

**Authors:** Fulvia Ferrazzi, Paola Sebastiani, Marco F Ramoni, Riccardo Bellazzi

**Affiliations:** 1 Dipartimento di Informatica e Sistemistica, Università degli Studi di Pavia, via Ferrata 1, 27100 Pavia, Italy; 2 Children's Hospital Informatics Program, Division of Health Sciences and Technology, Harvard Medical School and Massachusetts Institute of Technology, 300 Longwood Avenue, Boston MA 02115, USA; 3 Department of Biostatistics, Boston University School of Public Health, 715 Albany Street, Boston MA 02118, USA

## Abstract

**Background:**

Reverse engineering cellular networks is currently one of the most challenging problems in systems biology. Dynamic Bayesian networks (DBNs) seem to be particularly suitable for inferring relationships between cellular variables from the analysis of time series measurements of mRNA or protein concentrations. As evaluating inference results on a real dataset is controversial, the use of simulated data has been proposed. However, DBN approaches that use continuous variables, thus avoiding the information loss associated with discretization, have not yet been extensively assessed, and most of the proposed approaches have dealt with linear Gaussian models.

**Results:**

We propose a generalization of dynamic Gaussian networks to accommodate nonlinear dependencies between variables. As a benchmark dataset to test the new approach, we used data from a mathematical model of cell cycle control in budding yeast that realistically reproduces the complexity of a cellular system. We evaluated the ability of the networks to describe the dynamics of cellular systems and their precision in reconstructing the true underlying causal relationships between variables. We also tested the robustness of the results by analyzing the effect of noise on the data, and the impact of a different sampling time.

**Conclusion:**

The results confirmed that DBNs with Gaussian models can be effectively exploited for a first level analysis of data from complex cellular systems. The inferred models are parsimonious and have a satisfying goodness of fit. Furthermore, the networks not only offer a phenomenological description of the dynamics of cellular systems, but are also able to suggest hypotheses concerning the causal interactions between variables. The proposed nonlinear generalization of Gaussian models yielded models characterized by a slightly lower goodness of fit than the linear model, but a better ability to recover the true underlying connections between variables.

## Background

Reverse engineering cellular networks is one of the most challenging problems in systems biology. Starting with the measurements of certain variables, such as gene expression or protein concentration values, an attempt is made to infer the control mechanisms of the cellular system generating the available data, i.e. the underlying network of connections between its components. As only time series measurements provide information concerning the dynamics of a cell's regulatory mechanisms, recent studies have concentrated on analyzing such data.

The various reverse engineering methods proposed in the literature range from highly detailed models, such as those based on differential equations, to highly abstract models, such as Boolean networks. The former describe the molecular reactions taking place in a cell, and the latter represent cellular components as binary variables that are linked to each other by logical relationships [1,2]. Dynamic Bayesian networks (DBNs) are a special class of Bayesian networks (BNs) that model the stochastic evolution of a group of random variables over time, and offer a number of significant advantages over other methods [3-6]. Like BNs, when applied to cellular networks, DBNs describe cellular entities (i.e. mRNA or protein concentrations) by means of random variables and model the relationships between these variables both at qualitative and quantitative level [5]. At qualitative level, the relationships are encoded into a directed acyclic graph in which nodes represent the random variables, and arcs the conditional dependencies between them: for each node *x*, the *parents *of *x *are the variables that have a directed edge pointing to *x*. At quantitative level, the dependence relationships are described by means of conditional probability distributions. Because of their probabilistic framework, BNs and DBNs can automatically take into account the variability of biological systems, as well as the possible presence of experimental noise in the data.

However, while BNs only offer a static picture of the system, DBNs can show how variables regulate each other over time. For example, when analyzing gene expression data, BNs represent the expression level of each gene by a random variable, and infer a snapshot of the state of the cellular system at mRNA level. DBNs take this representation one step further, and represent the relationships between gene expression levels over time. Assuming a temporal dependency of order 1, one random variable is associated with the expression value of a gene at time *t*, and another with the expression level of the same gene at time *t *+ 1. In this representation, the direction of dependencies is constrained by the time dimension, and so the parents are the variables at time *t *and the children are the variables at time *t *+ 1. In this way, DBNs can also overcome the inability of BNs to represent feedback loops, due to the acyclicity constraint of the graph. This limitation makes BNs unsuitable for representing many biological systems in which feedback controls are a critical aspect of regulation.

The existing methods for learning BNs from observations can be adapted to DBNs. The selection of the best network to represent the data is treated as a Bayesian model selection problem, with different networks being compared by their posterior probability. This score makes a compromise between the ability of the inferred model to describe the data (i.e. its goodness of fit) and the number of parameters used. In this way, a more complex model is preferred over a simpler one only if its fitting ability significantly improves. The sound statistical framework of DBNs also allows them to incorporate prior knowledge and handle the possible presence of missing data and hidden variables (representing unobserved factors) in a principled way.

The formalism of dynamic Bayesian networks can be applied to describe the relationships between any type of cellular component, be it genes, proteins or other molecules, but most studies have so far concentrated on analyzing expression measurements generated by DNA microarrays (see for example [7-12]). These data provide a genome-wide view of cellular activity at transcription level, and this significant amount of information about the internal state of a cell can improve the chances of unraveling its control mechanisms. However, evaluating inference results on a real dataset is controversial. Validation of the connections obtained between the analyzed genes can be tried by searching the literature for known gene interactions, but the major disadvantage of this approach is that even if no supporting evidence for an inferred connection is found, it is not possible to conclude whether it is spurious or not without performing expensive and time-consuming experimental tests [13]. For this reason, the use of realistic simulated data was proposed and first applied by Smith *et al*. [14]. In this study, a complex biological system was simulated, taking into account various levels of organization from behavior to gene expression. Following this paradigm, detailed assessments of DBN inference algorithms applied to gene expression temporal data were made by Yu *et al*. [15] and Husmeier [13], but have some limitations in assessing the suitability of DBNs to analyze highly complex and nonlinear cellular systems. In order to simulate the data, Yu *et al*. used a simple model that does not describe the underlying molecular processes. Husmeier used a more realistic genetic network simulator consisting of a system of differential equations that describes gene interactions at the levels of transcription, translation and post-translational modifications [16] but, although it has also been recently used to test an extension of DBNs to incorporate perturbations [17], it only produces expression profiles for nine genes. Furthermore, the above-mentioned simulation studies concentrated on DBN approaches that require discretized expression data, but discretization can lead to a significant loss of information.

We therefore decided to concentrate on the class of DBNs known as *Gaussian networks*. These treat variables as continuous, and assume that the conditional distribution of each variable at time *t *+ 1 is Gaussian, with a mean that is a linear regression of the parent variables measured at time *t*.

As it is often argued that linear models are not suitable for capturing nonlinear dependencies between variables [10,13], we here propose a generalization of the linear Gaussian model in which parent values are transformed through the hyperbolic tangent function. In comparison with other approaches aimed at capturing nonlinear relationships [10], the proposed generalization retains the good computational efficiency of linear models.

In order to compare the performance of this nonlinear Gaussian model with the traditionally used linear model, we undertook a simulation study using data from a mathematical model of cell cycle control in budding yeast. This model, proposed by Chen *et al*. [18], contains 36 nonlinear differential equations and realistically reproduces the complexity of a cellular system. In particular, the questions we were interested in were:

• Are models inferred by means of Gaussian networks capable of fitting the data measured in cellular systems, and thus effectively describing their dynamics?

• Do Gaussian networks only provide a phenomenological description of the analyzed system, or are they also capable of learning the true underlying causal relationships between cellular variables?

• Do nonlinear Gaussian networks offer any advantages over linear networks in terms of the goodness of fit or reverse engineering capabilities?

The results are discussed in relation to these questions. We evaluate the goodness of fit of each inferred network (by calculating the root mean squared error), and its parsimony (the number of parameters used). We then quantitatively compare the inferred connections between the analyzed variables with their true relationships, and test the robustness of the results by analyzing the effect of noise on the data and the impact of a different sampling time.

## Results and discussion

The budding yeast cell cycle model by Chen *et al*. is described in the paper and at the authors' website [19], from which it is also possible to download files containing the model's equations and parameters that are ready to use with a simulator developed by G. Bard Ermentrout [20].

Chen *et al*. first created a literature-based wiring diagram for the cell cycle control mechanism in budding yeast (i.e. a graphic representation of the cellular components involved in the cell cycle and the reactions between them), then used the diagram to derive a mathematical model. Applying the general principles of biochemical kinetics, they converted the diagram into a set of 36 differential equations, plus some algebraic equations, which determine how the state of the control system (i.e. the vector of the concentrations of all its components) evolves over time, simulated the mathematical model, and showed that the solutions agree well with experimental data relating to various mutant strains of budding yeast. Almost all of the 36 variables represent protein concentrations (expressed using an arbitrary scale as the absolute concentrations of most of the proteins in the mechanism were not known at the time of publication), but some are auxiliary variables representing the mass and timing of cell cycle events. As the kinetic rates in the equations are low, the dynamics of the variable profiles are slow and comparable with those of gene expression temporal profiles. Using the nomenclature of Bayesian networks, the "parents" of each of the 36 variables are defined as the variables that appear in the differential equation describing its dynamics.

We simulated the data in the case of wild-type cells (using an integration step of 0.1 minutes) and sampled the values every five minutes, from time 0 to 100 min (about one cell cycle length). Our simulated dataset thus consists of 36 variables measured at 21 time points, a realistic number with respect to that typically used in temporal microarray experiments. The temporal profiles of each variable were standardized in order to have a mean value of zero and a standard deviation equal to one. The first validation was performed assuming noiseless sampling; the effect of the presence of noise on the simulated data was considered subsequently.

The aim of our study was to assess the ability of Gaussian networks in reverse engineering this differential equation model, which realistically describes the complex dynamics of a biological system. In particular, we were interested in comparing the traditionally used linear model with our proposed nonlinear generalization. In the former model, the conditional mean of a variable at time *t *+ 1 is a linear regression of the parent values at time *t *(see Equation (4) in Methods); in the latter, the parent values are first transformed by means of the hyperbolic tangent function Φ(*x*) = *tanh*(*αx*) (see Equation (5) in Methods). Thus, this model is nonlinear with respect to the parent values, but it is still linear in the regression parameters. We examined different values for the parameter *α*, namely *α *= {0.4, 0.6, 0.8, 1, 1.2, 1.4, 1.6, 1.8, 2, 2.5, 3, 3.5, 4, 6, 8, 10}.

We first examined the fitting and parsimony of the inferred models. For each model, the goodness of fit is represented by the *root mean squared error *(RMSE), calculated as the average of the root mean squared errors relating to each variable (RMSE_*i*_). Given *v *variables and *T *time points, we have:

RMSE=1v∑i=1vRMSEi
 MathType@MTEF@5@5@+=feaafiart1ev1aaatCvAUfKttLearuWrP9MDH5MBPbIqV92AaeXatLxBI9gBaebbnrfifHhDYfgasaacH8akY=wiFfYdH8Gipec8Eeeu0xXdbba9frFj0=OqFfea0dXdd9vqai=hGuQ8kuc9pgc9s8qqaq=dirpe0xb9q8qiLsFr0=vr0=vr0dc8meaabaqaciaacaGaaeqabaqabeGadaaakeaacqWGsbGucqWGnbqtcqWGtbWucqWGfbqrcqGH9aqpdaWcaaqaaiabigdaXaqaaiabdAha2baadaaeWbqaaiabdkfasjabd2eanjabdofatjabdweafnaaBaaaleaacqWGPbqAaeqaaaqaaiabdMgaPjabg2da9iabigdaXaqaaiabdAha2bqdcqGHris5aaaa@41CF@

**Figure 1:**
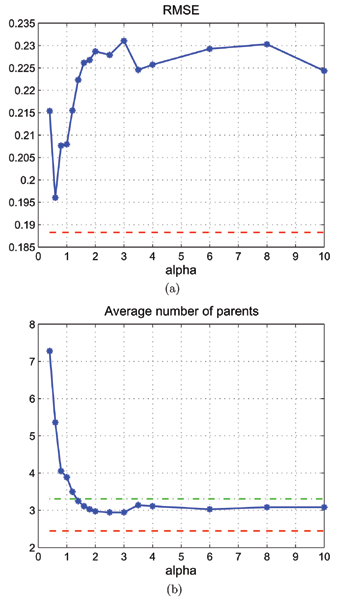
**Goodness of fit and parsimony of the inferred models**. Plots of the RMSE (a) and average number of parents per variable (b) for the nonlinear models, as a function of the parameter *α *of the hyperbolic tangent function (continuous blue curves); the dashed red curves refer to the RMSE and average number of parents for the linear regression model. The dash-dotted green curve in (b) represents the average number of parents in the differential equation model (i.e. the average number of true parents). Further analyses showed that, for *α *→ + ∞, the RMSE saturates at 0.247, and the average number of parents saturates at 3.4.

**Figure 2:**
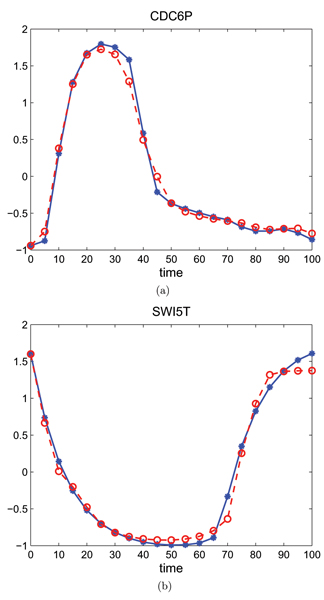
**Examples of fitted profiles**. Plots of the observed (continuous blue line) and fitted profile (dashed red line) for two analyzed variables, obtained using the nonlinear models corresponding to *α *= 0.6 (a) and *α *= 2 (b). Time is expressed in minutes.

RMSEi=1T−1∑t=2T(yit−y^it)2
 MathType@MTEF@5@5@+=feaafiart1ev1aaatCvAUfKttLearuWrP9MDH5MBPbIqV92AaeXatLxBI9gBaebbnrfifHhDYfgasaacH8akY=wiFfYdH8Gipec8Eeeu0xXdbba9frFj0=OqFfea0dXdd9vqai=hGuQ8kuc9pgc9s8qqaq=dirpe0xb9q8qiLsFr0=vr0=vr0dc8meaabaqaciaacaGaaeqabaqabeGadaaakeaacqWGsbGucqWGnbqtcqWGtbWucqWGfbqrdaWgaaWcbaGaemyAaKgabeaakiabg2da9maakaaabaWaaSaaaeaacqaIXaqmaeaacqWGubavcqGHsislcqaIXaqmaaWaaabCaeaacqGGOaakcqWG5bqEdaWgaaWcbaGaemyAaKMaemiDaqhabeaakiabgkHiTiqbdMha5zaajaWaaSbaaSqaaiabdMgaPjabdsha0bqabaGccqGGPaqkdaahaaWcbeqaaiabikdaYaaaaeaacqWG0baDcqGH9aqpcqaIYaGmaeaacqWGubava0GaeyyeIuoaaSqabaaaaa@4B97@

where *y*_*it *_is the observed (in this case: simulated) value for variable *i *at time *t*, and y^
 MathType@MTEF@5@5@+=feaafiart1ev1aaatCvAUfKttLearuWrP9MDH5MBPbIqV92AaeXatLxBI9gBaebbnrfifHhDYfgasaacH8akY=wiFfYdH8Gipec8Eeeu0xXdbba9frFj0=OqFfea0dXdd9vqai=hGuQ8kuc9pgc9s8qqaq=dirpe0xb9q8qiLsFr0=vr0=vr0dc8meaabaqaciaacaGaaeqabaqabeGadaaakeaacuWG5bqEgaqcaaaa@2E37@_*it *_is the corresponding predicted value, assumed to be equal to the expected value of *Y*_*i *_given the parent values at the previous time point. Parsimony is represented by the average of the number of parents inferred for each variable. Figure 1 shows how the RMSE and average number of parents vary in relation to the values of *α *in the nonlinear model (continuous blue curves). The dashed red curves represent the results for the linear regression model. The linear model seems to be characterized by a better goodness of fit (the RMSE is smaller) and to be more parsimonious (the average number of parents is lower). However, it is necessary to note that the fitting is satisfactory for all the examined models: for example, Figure 2 shows the observed and fitted profiles of two variables in the cases *α *= 0.6 and *α *= 2. Moreover, the parsimony of the nonlinear models is comparable with that of the differential equation model used to simulate the data: in this model, the total number of parent-child relationships is 119, which corresponds to an average of 3.3 parents per variable.

The other aim of our study was to make a quantitative assessment of the ability of Gaussian networks to learn the causal interactions between the analyzed variables. To this end, we compared the "true parents" of each variable (i.e. its parents in the differential equation model) with the parents found using the DBN algorithm by calculating the *recall *(R) and the *precision *(P):

R=TPTP+FNP=TPTP+FP     (1)
 MathType@MTEF@5@5@+=feaafiart1ev1aaatCvAUfKttLearuWrP9MDH5MBPbIqV92AaeXatLxBI9gBaebbnrfifHhDYfgasaacH8akY=wiFfYdH8Gipec8Eeeu0xXdbba9frFj0=OqFfea0dXdd9vqai=hGuQ8kuc9pgc9s8qqaq=dirpe0xb9q8qiLsFr0=vr0=vr0dc8meaabaqaciaacaGaaeqabaqabeGadaaakeaafaqabeqacaaabaGaemOuaiLaeyypa0ZaaSaaaeaacqWGubavcqWGqbauaeaacqWGubavcqWGqbaucqGHRaWkcqWGgbGrcqWGobGtaaaabaGaemiuaaLaeyypa0ZaaSaaaeaacqWGubavcqWGqbauaeaacqWGubavcqWGqbaucqGHRaWkcqWGgbGrcqWGqbauaaaaaiaaxMaacaWLjaWaaeWaaeaacqaIXaqmaiaawIcacaGLPaaaaaa@449C@

*TP *(true positives) is the number of inferred parents that are "true parents", *FP *(false positives) the number of erroneously inferred parents, and *FN *(false negatives) the number of "true parents" that are not recovered. R therefore corresponds to the fraction of "true parents" correctly inferred by the DBN algorithm, while P is the fraction of inferred parents that are also "true parents".

R and P can be summarized using their harmonic mean, called *F-measure *(F), which tends to be closer to the minimum between the two:

F=21R+1P=2RPR+P     (2)
 MathType@MTEF@5@5@+=feaafiart1ev1aaatCvAUfKttLearuWrP9MDH5MBPbIqV92AaeXatLxBI9gBaebbnrfifHhDYfgasaacH8akY=wiFfYdH8Gipec8Eeeu0xXdbba9frFj0=OqFfea0dXdd9vqai=hGuQ8kuc9pgc9s8qqaq=dirpe0xb9q8qiLsFr0=vr0=vr0dc8meaabaqaciaacaGaaeqabaqabeGadaaakeaacqWGgbGrcqGH9aqpdaWcaaqaaiabikdaYaqaamaalaaabaGaeGymaedabaGaemOuaifaaiabgUcaRmaalaaabaGaeGymaedabaGaemiuaafaaaaacqGH9aqpdaWcaaqaaiabikdaYiabdkfasjabdcfaqbqaaiabdkfasjabgUcaRiabdcfaqbaacaWLjaGaaCzcamaabmaabaGaeGOmaidacaGLOaGaayzkaaaaaa@4056@

Two other commonly used measures to assess an algorithm's performance are sensitivity (which coincides with recall) and specificity:

specificity=TNFP+TN     (3)
 MathType@MTEF@5@5@+=feaafiart1ev1aaatCvAUfKttLearuWrP9MDH5MBPbIqV92AaeXatLxBI9gBaebbnrfifHhDYfgasaacH8akY=wiFfYdH8Gipec8Eeeu0xXdbba9frFj0=OqFfea0dXdd9vqai=hGuQ8kuc9pgc9s8qqaq=dirpe0xb9q8qiLsFr0=vr0=vr0dc8meaabaqaciaacaGaaeqabaqabeGadaaakeaacqWGZbWCcqWGWbaCcqWGLbqzcqWGJbWycqWGPbqAcqWGMbGzcqWGPbqAcqWGJbWycqWGPbqAcqWG0baDcqWG5bqEcqGH9aqpdaWcaaqaaiabdsfaujabd6eaobqaaiabdAeagjabdcfaqjabgUcaRiabdsfaujabd6eaobaacaWLjaGaaCzcamaabmaabaGaeG4mamdacaGLOaGaayzkaaaaaa@486A@

*TN *(true negatives) is the number of "negatives" (or missing interactions between the analyzed variables) that are also not present in the inferred network. We chose to use precision instead of specificity because the very high number of "negatives" makes specificity an unsuitable measure of performance, as also pointed out in [21]. Assuming that we are analyzing *N *variables, the total number of possible binary interactions is *N*^2^, but the actual number *k *of interactions is normally much lower than *N*^2 ^because of the sparsity of cellular networks. Thus, the denominator of specificity (i.e. the total number of "negatives" [*FP *+ *TN *= *N*^2 ^- *k*]) is very high and even slight differences from 1 in specificity correspond to a large number of false positives. Precision also has the advantage that it can be interpreted as the "expected success rate in the experimental validation of predicted interactions" [21].

**Figure 3:**
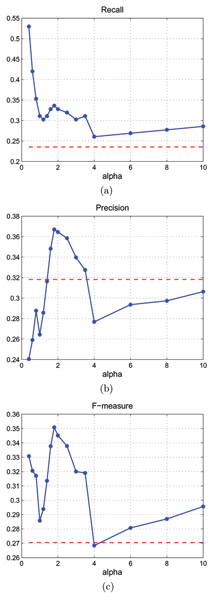
**Comparison of the inferred connections and true relationships between the analyzed variables**. Plots of recall (a), precision (b) and F-measures (c) of the nonlinear models as a function of the parameter *α *of the hyperbolic tangent function (continuous blue curves). The dashed red curves refer to the recall, precision and F-measure of the linear regression model. Further analyses showed that, for *α *→ + ∞, recall saturates at 0.27, precision at 0.26, and the F-measure at 0.26.

Figure 3 shows how R, P and F vary in relation to the values of *α *for the nonlinear model (continuous blue curves). Once again the dashed red curves represent the results of the linear model.

**Figure 4:**
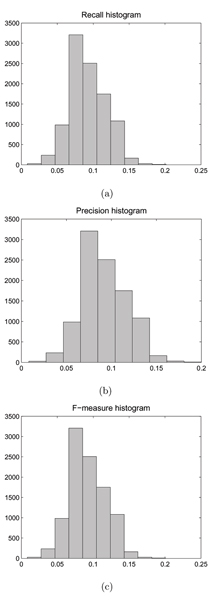
**Random networks**. Histograms of recall (a), precision (b) and F-measures (c) for 10^4 ^random networks containing 120 links (equal to the number of parent-child relationships in the true model).

It is important to underline that the R and P (and therefore F) of the networks learned with the DBN algorithm are significantly higher than those obtainable if the network structures are randomly created. This can be clearly seen in Figure 4, which shows the histogram of the R, P and F values for 10^4 ^networks created by assigning at random 120 links (the number of parent-child relationships in the true model) of the possible 36^2^.

Alternatively, in order to assess the significance of the inferred networks, it is also possible to calculate their *p-values *as proposed by Dojer *et al*. [17]: the p-value of a network with *h *true out of *M *inferred edges is defined as the probability of finding at least *h *true edges when choosing *M *edges at random. This probability can be calculated using hypergeometric distribution and, in our case, the p-values were all less than 10^-9^.

It is interesting to look at the best network model in terms of goodness of fit, and the best model in terms of its ability to recover the causal relationships between variables. The former (i.e. the model with the lowest RMSE [0.196]) corresponds to *α *= 0.6, and has F = 0.321; the latter (i.e. the model with the highest F [0.351]) corresponds to *α *= 1.8 and has RMSE = 0.227. Different *α *values can thus slightly favor the goodness of fit or accuracy of the inferred models.

Moreover, comparison of the linear regression and nonlinear model showed that the latter performs better at recovering the causal connections between variables (R is higher, and so is F). For example, the recall of the above model corresponding to *α *= 1.8 is 43% higher than that of the linear model (0.336 vs. 0.235): this corresponds to 40 out of 119 true links recovered, instead of 28. In addition, its precision is 15% higher (0.367 vs. 0.318), thus leading to a 30% improvement in F (0.351 vs. 0.271).

### Sensitivity analysis in the presence of noise

When dealing with experimental data such as gene expression measurements from DNA microarrays, the presence of a certain level of noise is unavoidable. As mentioned above, one advantage of the Bayesian network approach is that it can naturally take into account the effect of the presence of noise on the data, but it is nonetheless interesting to assess the robustness of the results quantitatively. To this end, we added noise with a constant coefficient of variation (*CV*) to the simulated profiles. In particular, for every profile at each time point, we added a random variable extracted from a Gaussian distribution with zero mean and standard deviation *σ *= *CV abs*(y¯
 MathType@MTEF@5@5@+=feaafiart1ev1aaatCvAUfKttLearuWrP9MDH5MBPbIqV92AaeXatLxBI9gBaebbnrfifHhDYfgasaacH8akY=wiFfYdH8Gipec8Eeeu0xXdbba9frFj0=OqFfea0dXdd9vqai=hGuQ8kuc9pgc9s8qqaq=dirpe0xb9q8qiLsFr0=vr0=vr0dc8meaabaqaciaacaGaaeqabaqabeGadaaakeaacuWG5bqEgaqeaaaa@2E3F@) to the simulated value y¯
 MathType@MTEF@5@5@+=feaafiart1ev1aaatCvAUfKttLearuWrP9MDH5MBPbIqV92AaeXatLxBI9gBaebbnrfifHhDYfgasaacH8akY=wiFfYdH8Gipec8Eeeu0xXdbba9frFj0=OqFfea0dXdd9vqai=hGuQ8kuc9pgc9s8qqaq=dirpe0xb9q8qiLsFr0=vr0=vr0dc8meaabaqaciaacaGaaeqabaqabeGadaaakeaacuWG5bqEgaqeaaaa@2E3F@. The values considered for the coefficient of variation were *CV *= {0.05, 0.1, 0.2, 0.3}, which means that the standard deviation of the noise was respectively 5, 10, 20 and 30% of the simulated values. The noisy profiles were analyzed using both the linear regression and the nonlinear model, and their performances were assessed as described above by considering the RMSE and average number of parents on one hand, and recall, precision and F-measures on the other.

**Figure 5:**
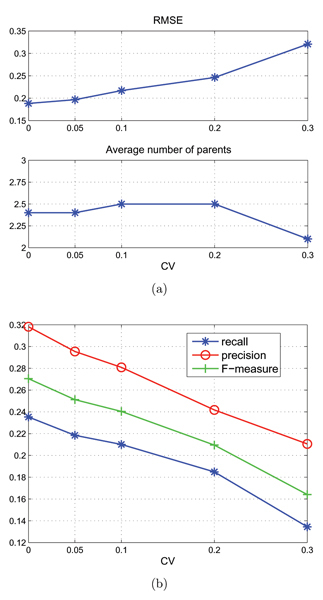
**Robustness of the linear model in the presence of noise**. Plots of the RMSE and average number of parents (a), and recall, precision and F-measures (b) of the linear model as a function of the *CV *of the noise. *CV *= 0 corresponds to the noiseless case.

Comparison of the results of the linear model and those in the noiseless case revealed slight variations in the parsimony of the inferred models (average number of parents), but a worse goodness of fit. As could be expected, the RMSE increased in proportion to the increasing levels of noise (higher *CV*). In terms of the ability to recover true connections, recall slightly decreased in the case of *CV *= 0.05 and *CV *= 0.1, but significantly worsened in the case *CV *= 0.3. The precision for every *CV *value was less than in the noiseless case and, consequently, so was the F-measure. These results are summarized in Figure 5, which shows that F is around 24% for *CV *= {0.05, 0.1} and becomes 16% for *CV *= 0.3.

**Figure 6:**
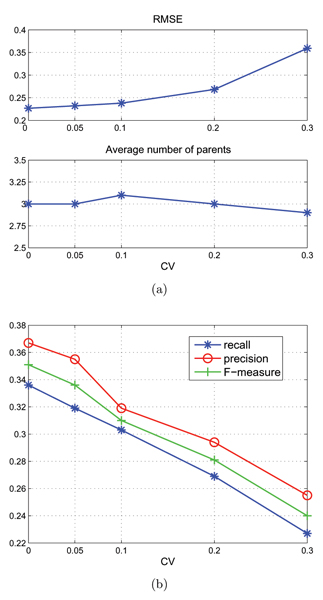
**Robustness of the nonlinear model in the presence of noise**. Plots of the RMSE and average number of parents (a), and recall, precision and F-measures (b) of the nonlinear model corresponding to *α *= 1.8, as a function of the *CV *of the noise. *CV *= 0 corresponds to the noiseless case.

The results of the nonlinear model showed that, in correspondence with every value of the parameter *α*, its parsimony is comparable with that of the noiseless case. As with the linear model, the RMSE increased constantly as *CV *increased. With a few exceptions, the recall corresponding to each *α *value tended to decrease as the *CV *increased, as did precision and, therefore, the F-measure. However, in general, F remained about 30% or more for *CV *= {0.05, 0.1}, and was not less than 23% even when *CV *= 0.3. The decrease in performance thus seems to be less significant than that relating to the linear model. Figure 6 shows the results for *α *= 1.8, which is the value with the best F (F = 0.351).

### Sensitivity analysis varying the sampling interval

Previous biological knowledge of the length of the cell cycle in the examined system allowed us to restrict our analysis to this time frame. We expected that the more time points we collected (i.e. the smaller the chosen sampling interval), the better the performance of our reverse engineering algorithm would be. However, experimental measurements are often expensive and/or difficult and this is the main reason why biological temporal profiles usually contain few time points. Thus, it is interesting to make a quantitative assessment of the extent to which the performance of the algorithm depends on the sampling interval in order to have some indications concerning the minimum number of time points necessary to obtain satisfactory results.

Our previous analyses had always used a sampling interval of five minutes and so, once again using the simulated data from time 0 to 100 min (about one cell cycle length) in the case of wild-type cells, we sampled values at intervals *s *= {1, 2, 10} minutes, thus producing datasets with respectively {101, 51, 11} time points. This enabled us to examine how the results vary with a larger or smaller number than our baseline of 21 points.

**Figure 7:**
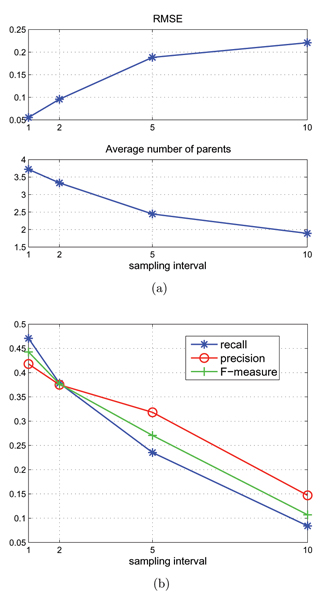
**Robustness of the linear model when varying the sampling interval**. Plots of the RMSE and average number of parents (a), and recall, precision and F-measures (b) of the linear model, as a function of the sampling interval *s *(in minutes).

The results for the linear regression model, considered together with those obtained with our baseline of *s *= 5, showed that the average number of parents decreases as *s *increases. This is probably due to the fact that the addition of parents does not significantly improve the fitting for higher values of *s*, and so the Bayesian score does not improve. The RMSE was very low at *s *= {1, 2}, and increased constantly as *s *increased, whereas recall and precision constantly decreased: F went from 44% at *s *= 1 to 27% at *s *= 5, and there was a sharp decline in performance at *s *= 10, when F was about 11%. The results are summarized in Figure 7.

**Figure 8:**
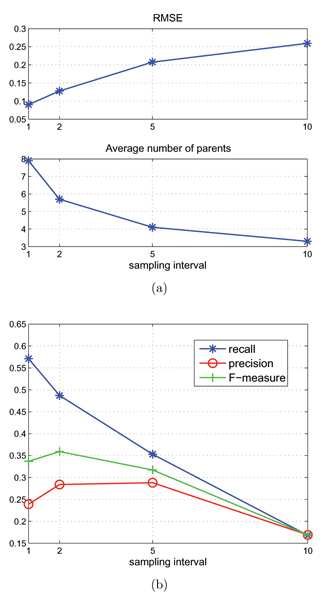
**Robustness of the nonlinear model when varying the sampling interval**. Plots of the RMSE and average number of parents (a), and recall, precision and F-measures (b) of the nonlinear model corresponding to *α *= 0.8, as a function of the sampling interval *s *(in minutes).

In the case of the nonlinear model, and considering each value of *α*, the average number of parents also decreased as *s *increased. The RMSE was very low at *s *= {1, 2}, and became higher with longer sampling intervals. Recall decreased as *s *increased, while precision was greatest at *s *= 5 (in most cases) or *s *= 2 (two cases). There thus seems to be a compromise between recall (best values at *s *= 1) and precision. With *α *< 2, F is more than 27% (most frequently more than 30%) at *s *= {1, 2, 5}, and becomes 17 – 20% at *s *= 10. With *α *≥ 2, F is always more than 20%. Figure 8 shows the results for *α *= 0.8, the value with the best F (F = 0.359).

The results thus show that the goodness of fit of the models worsens as the sampling interval increases, whereas the ability of the algorithm to recover the true causal connections between variables is best at *s *= 2 or *s *= 5. Moreover, the decline in performance at *s *= 10 is much less than in the case of the linear model.

### Comparison with other published simulated studies

It is interesting to compare the results obtained in our study with those of published simulation studies of DBNs with discrete variables.

The model used by Husmeier simulates expression time series for a network of nine connected genes, to which another 41 unconnected randomly up- or down-regulated genes were added for a total of 50 genes. The results were evaluated using sensitivity and complementary specificity (1-specificity) rather than recall and precision. The high sensitivity values obtained by the author in some trials performed must always be carefully assessed together with their complementary specificity because, as explained above, even very low values of complementary specificity (and thus high values of specificity) can correspond to a significant number of false positives. For example, Husmeier himself stressed that the whole set of true connections can be recovered only at a complementary specificity of 75%, which corresponds to an extremely high number of spurious connections. Moreover, an example network shown by Husmeier in relation to only the nine connected nodes has a recall (sensitivity) of 36% and similar precision: these figures are comparable to those obtained in our study.

Yu *et al*. used a linear model to generate data for 10 networks, each containing 20 nodes. Between eight and 12 nodes are connected in each network, whereas the others are unconnected and move in a random walk. Recall and imprecision (1-precision) are used to assess the algorithm's performance. The authors present most results for a much higher number of data points than ours (up to 2000), and show that it is possible to obtain high values of recall and precision only in the presence of more than one hundred points. With 2000 points, they obtained a recall of 90% and a precision of almost 100% (F = 95%); for 300 points, recall decreased to about 50 – 55% and precision to 85% (F = 67%); for 100 points, recall was still about 50% and precision was similar (F = 50%); but with 25 points recall decreased to 30 – 35% and precision to 10% (F = 16%).

As mentioned above, our analysis concentrated on short time series because temporal microarray experiments do not typically contain more than a few tens of time points, and so the results of our study should better approximate the recall and precision obtainable when analyzing real gene expression data. The above recall and precision values show that results obtained by Yu *et al*. for short time series are not significantly different from ours, although they simulated data using a simpler model.

Nonetheless, it is interesting to investigate the performance of our method in the presence of longer time series, and particularly interesting to compare our results with those of Yu *et al.*, who kindly made the simulator they used to generate their data available to us. This simulator produces profiles with continuous values, which Yu *et al*. need to discretize in order to apply their DBN algorithm.

For each of the 10 networks used by Yu *et al*. in their study, we simulated one dataset with 100 points and another with 300, using a sampling interval of 5. The temporal profiles of each variable were standardized before the analysis with our algorithm.

Table 1 shows the recall, precision and F-measure obtained using the linear model and the nonlinear model corresponding to *α *= 1. For each number of time points (100 or 300), the values shown are averaged over the 10 datasets simulated in correspondence with the different network structures. As can be seen, with both 100 and 300 time points, the recall obtained with Gaussian networks is greater than that reported by Yu *et al.*, the precision is comparable or slightly lower, and the synthetic F-measure is always higher. In this case, the precision of the linear model outperforms that of the nonlinear model using hyperbolic tangent functions. This may be because the simulator used by Yu *et al*. is based on a dynamical system which is linear over a wide range of variable values.

It can therefore be said that Gaussian networks seem to have advantages over discrete variable networks if a limited amount of data is available as they do not suffer from information loss due to discretization, and are more parsimonious than discrete models. In the case of discrete models, the number of parameters required to describe the dependence of a variable on its parents depends on the number of possible combinations of the parent values: i.e. assuming a binary variable with three parents that can each have two possible values, 2^3 ^= 8 parameters are required. On the contrary, in Gaussian networks, each parent corresponds to one parameter in the regression equation, and so only three parameters are required for three parents (or four if a constant parameter is also used). The reduction in the number of required parameters becomes more obvious as the number of parents or the number of discretization categories increases.

## Conclusion

We propose a generalization of dynamic Gaussian networks as a means of better capturing the nonlinearity of the relationships between cellular variables, in which the parent values are transformed using the hyperbolic tangent function Φ(*x*) = *tanh*(*αx*). In order to compare the performance of this approach with that of traditional linear Gaussian networks, we undertook a novel simulation study using data from a differential equation model proposed by Chen *et al.*, which reproduces well the complexity and nonlinearity of cell cycle control mechanisms in budding yeast [18].

We simulated data in the case of wild-type cells by sampling the values every five minutes from time 0 to 100 minutes (about one cell cycle length), thus obtaining a dataset of 36 variables measured at 21 time points. The results show that the linear model has a better goodness of fit and is slightly more parsimonious, whereas the nonlinear model performs better at recovering the true underlying causal relationships between variables: the F-measure (a summary of recall and precision) has a maximum value of 35% and is never less than that of the linear model (27%). The figures for both models are significantly higher than those obtainable if the network structures are randomly created.

We performed a sensitivity analysis in the presence of data affected by Gaussian noise with a constant coefficient of variation (*CV*), and found that the parsimony of both models is comparable with that observed in the noiseless case, whereas the goodness of fit worsens as the *CV *increases. The ability to recover causal connections also decreases, although the decline in the performance of the nonlinear model is less significant than that of the linear model: in the former, F is never less than 23% even with *CV *= 0.3 whereas, in the latter, it becomes about 16%.

We also assessed the performance of the proposed models using sampling times other that our baseline of 5 minutes, i.e. *s *= {1, 2, 10} minutes, corresponding to datasets with respectively {101, 51, 11} time points. At *s *= {1, 2} min, the performance of the linear model improves in terms of fitting accuracy and the ability to recover true relationships; at *s *= 10 min, performance significantly decreases, thus showing that 11 time points are too few to recover the network of connections between the analyzed variables. On the contrary, in the case of the nonlinear model, although fitting accuracy and parsimony improve with more time points, the best compromise between recall and precision often occurs at our baseline of *s *= 5 min. These results indicate that, in the (unlikely) case in which many time points are available, the linear model is better at recovering the causal interactions between variables (F = 44% at *s *= 1). However, in the presence of a number of time points similar to that most often available in biological time series measurements, the use of the nonlinear model is advantageous, as seen in the case of *s *= 5.

Overall, our findings confirm that DBNs with Gaussian models can be effectively used for the first level analysis of data from complex cellular systems because they not only offer a phenomenological description of the system dynamics, but also suggest hypotheses concerning the causal relationships between variables. However, given the significant number of inferred false positive interactions, these hypotheses need to be verified by subsequent biological validation.

The proposed generalization of Gaussian models generally yielded models that were characterized by a slightly lower goodness of fit than the linear model, but a better ability to recover the true connections between variables. This advantage was also maintained in different error models and seemed to be particularly significant in the presence of a limited number of time points. The results thus suggest that, if the main objective of a study is to have a model with good predictive ability, the simpler linear Gaussian model is advantageous but, if the objective is to infer causal relationships between variables, it is necessary to move toward nonlinear functions. It is worth mentioning that one issue related to the use of nonlinear functions of parent variables is that standard probabilistic algorithms for inference do not apply. However, it is always possible to perform inference using Gibbs sampling.

The proposed Gaussian network approach is a valuable modeling tool. It is more parsimonious than discrete models because fewer parameters need to be estimated from the data. At the same time, thanks to the possibility of exploiting the nonlinear functions of the parent values, it is flexible enough to be used in a large variety of applications.

Given these promising results, we would like to extend our investigation by evaluating the use of different nonlinear functions Φ(·). Other very interesting extensions already pursued by some researchers include the introduction of prior knowledge in the learning process, and the integration of different types of data [11,22-24]. Each high-throughput technology can offer only a partial view of the highly nonlinear dynamical processes that take place in a cell, whereas the combination of knowledge and data from different sources should lead to a more profound understanding.

## Methods

### Gaussian networks

A DBN is defined by a directed acyclic graph in which the nodes represent stochastic variables and the directed arcs represent the temporal dependencies between them, which are quantified by conditional probability distributions.

Given a database of measurements for *v *variables (eg. genes or proteins) at *T *consecutive and equally spaced time points, the values of the variables at time *t *are represented by the random variables *Y*(*t*) = {*Y*_1_(*t*), *Y*_2_(*t*),...,*Y*_*v*_(*t*)}. In order to derive the DBN encoding the dependencies over the random variables *Y *at the different time points, it is assumed that the process under study (the dynamics of our system) is

• *Markovian *[i.e. *p*(*Y*(*t *+ 1)|*Y*(0),...,*Y*(*t*)) = *p*(*Y*(*t *+ 1)|*Y*(*t*))] and

• *time homogeneous *[the transition probability *p*(*Y*(*t *+ 1)|*Y*(*t*)) is independent of *t*]

Given these assumptions, we only need to learn the transition network between the variables at time *t *and time *t *+ 1 [25]. To this end, it is necessary to choose a probability model and a search strategy.

#### Probability model

Linear Gaussian networks assume that the variables *Y*_1_,...,*Y*_*v *_are all continuous, and that the conditional distribution of each variable *Y*_*i *_given its parents *Pa*(*y*_*i*_) = (*Y*_*i*1_,...,*Y*_*ip*(*i*)_) follows a Gaussian distribution with mean *μ*_*i *_and conditional variance σi2
 MathType@MTEF@5@5@+=feaafiart1ev1aaatCvAUfKttLearuWrP9MDH5MBPbIqV92AaeXatLxBI9gBaebbnrfifHhDYfgasaacH8akY=wiFfYdH8Gipec8Eeeu0xXdbba9frFj0=OqFfea0dXdd9vqai=hGuQ8kuc9pgc9s8qqaq=dirpe0xb9q8qiLsFr0=vr0=vr0dc8meaabaqaciaacaGaaeqabaqabeGadaaakeaaiiGacqWFdpWCdaqhaaWcbaGaemyAaKgabaGaeGOmaidaaaaa@30F0@ = 1/*τ*_*i *_[6]. The parameter *τ*_*i *_is called precision. The mean *μ*_*i *_is typically the linear regression function of the parent variables and regression parameters {*β*_*i*0_, *β*_*i*1_,...,*β*_*ip*(*i*)_} as in the equation

μi=βi0+∑j=1p(i)βijyij     (4)
 MathType@MTEF@5@5@+=feaafiart1ev1aaatCvAUfKttLearuWrP9MDH5MBPbIqV92AaeXatLxBI9gBaebbnrfifHhDYfgasaacH8akY=wiFfYdH8Gipec8Eeeu0xXdbba9frFj0=OqFfea0dXdd9vqai=hGuQ8kuc9pgc9s8qqaq=dirpe0xb9q8qiLsFr0=vr0=vr0dc8meaabaqaciaacaGaaeqabaqabeGadaaakeaaiiGacqWF8oqBdaWgaaWcbaGaemyAaKgabeaakiabg2da9iab=j7aInaaBaaaleaacqWGPbqAcqaIWaamaeqaaOGaey4kaSYaaabCaeaacqWFYoGydaWgaaWcbaGaemyAaKMaemOAaOgabeaakiabdMha5naaBaaaleaacqWGPbqAcqWGQbGAaeqaaaqaaiabdQgaQjabg2da9iabigdaXaqaaiabdchaWjabcIcaOiabdMgaPjabcMcaPaqdcqGHris5aOGaaCzcaiaaxMaadaqadaqaaiabisda0aGaayjkaiaawMcaaaaa@4CB3@

that models the conditional mean of *Y*_*i *_at time *t *+ 1 given the parent values *y*_*ij*_, measured at time *t*.

The use of linear models makes the inference process computationally amenable even with hundreds of variables. However, traditional linear regression models may be inappropriate when there are nonlinear relationships between variables. In this paper we also propose a generalization of the linear regression model described in Equation (4). In this generalization the dependency of each variable on its parents is described as a linear combination of nonlinear functions Φ(·) of the parent values:

μi=βi0+∑j=1p(i)βijΦ(yij)     (5)
 MathType@MTEF@5@5@+=feaafiart1ev1aaatCvAUfKttLearuWrP9MDH5MBPbIqV92AaeXatLxBI9gBaebbnrfifHhDYfgasaacH8akY=wiFfYdH8Gipec8Eeeu0xXdbba9frFj0=OqFfea0dXdd9vqai=hGuQ8kuc9pgc9s8qqaq=dirpe0xb9q8qiLsFr0=vr0=vr0dc8meaabaqaciaacaGaaeqabaqabeGadaaakeaaiiGacqWF8oqBdaWgaaWcbaGaemyAaKgabeaakiabg2da9iab=j7aInaaBaaaleaacqWGPbqAcqaIWaamaeqaaOGaey4kaSYaaabCaeaacqWFYoGydaWgaaWcbaGaemyAaKMaemOAaOgabeaakiabfA6agjabcIcaOiabdMha5naaBaaaleaacqWGPbqAcqWGQbGAaeqaaOGaeiykaKIaaCzcaiaaxMaadaqadaqaaiabiwda1aGaayjkaiaawMcaaaWcbaGaemOAaOMaeyypa0JaeGymaedabaGaemiCaaNaeiikaGIaemyAaKMaeiykaKcaniabggHiLdaaaa@4FEC@

**Figure 9:**
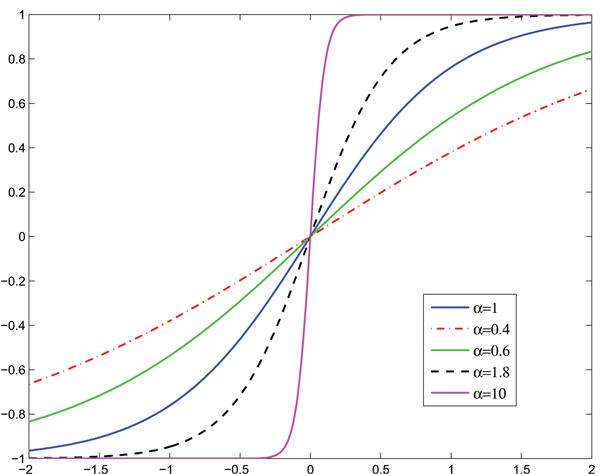
**Hyperbolic tangent function**. Plot of the hyperbolic tangent function y = tanh(*α*x) for different values of *α*. The function approaches a step function at increasingly high values of *α*.

This model is known as a nonlinear expansion of the input data. As it is reasonable to assume that the rate of the production/elimination of mRNA or proteins cannot indefinitely grow, we set Φ(·) equal to the hyperbolic tangent function Φ(*x*) = *tanh*(*αx*), where *α *is a predefined parameter. Figure 9 shows the different shapes of the function for different values of *α*. Note that the function approaches a step function for larger values of *α*. It is important to note that the parameterization of the mean function in Equation (5) is still linear in the regression parameters, for known *α*, and so the learning process is scalable to hundreds of variables as in the case of model (4),

#### Scoring metric

To induce the DBN from data we use the Bayesian model selection procedure and search for the network with maximum posterior probability given the data. By Bayes' theorem, the posterior probability of a network *M*_*h *_given data *D *is

*p*(*M*_*h*_|*D*)∝ *p*(*M*_*h*_)*p*(*D*|*M*_*h*_)     (6)

where *p*(*M*_*h*_) is the prior probability of the network and *p*(*D*|*M*_*h*_) is the marginal likelihood. The marginal likelihood is the solution of the integral

p(D|Mh)=∫p(D|θh,Mh)p(θh|Mh)dθh     (7)
 MathType@MTEF@5@5@+=feaafiart1ev1aaatCvAUfKttLearuWrP9MDH5MBPbIqV92AaeXatLxBI9gBaebbnrfifHhDYfgasaacH8akY=wiFfYdH8Gipec8Eeeu0xXdbba9frFj0=OqFfea0dXdd9vqai=hGuQ8kuc9pgc9s8qqaq=dirpe0xb9q8qiLsFr0=vr0=vr0dc8meaabaqaciaacaGaaeqabaqabeGadaaakeaacqWGWbaCcqGGOaakcqWGebarcqGG8baFcqWGnbqtdaWgaaWcbaGaemiAaGgabeaakiabcMcaPiabg2da9maapeaabaGaemiCaaNaeiikaGIaemiraqKaeiiFaWhcciGae8hUde3aaSbaaSqaaiabdIgaObqabaGccqGGSaalcqWGnbqtdaWgaaWcbaGaemiAaGgabeaakiabcMcaPiabdchaWjabcIcaOiab=H7aXnaaBaaaleaacqWGObaAaeqaaOGaeiiFaWNaemyta00aaSbaaSqaaiabdIgaObqabaGccqGGPaqkcqWGKbazcqWF4oqCdaWgaaWcbaGaemiAaGgabeaaaeqabeqdcqGHRiI8aOGaaCzcaiaaxMaadaqadaqaaiabiEda3aGaayjkaiaawMcaaaaa@5771@

in which *p*(*D*|*θ*_*h*_, *M*_*h*_) is the likelihood of the data given the model *M*_*h *_and the vector of parameters *θ*_*h*_, and *p*(*θ*_*h*_|*M*_*h*_) is the prior density of the parameters. By averaging out the parameters, the marginal likelihood provides a measure of the likelihood of the model regardless of the specific values of the parameters.

By the *Local Markov Property *that states each variable is independent of its non-descendants given its parents, the marginal likelihood *p*(*D*|*M*_*h*_) can be factorized into the product of the factors *p*(*D*|*M*_*hi*_) that represent the marginal likelihood of the dependency of each variable *Y*_*i *_at time *t *+ 1 on its parents at time *t*:

p(D|Mh)=∏ip(D|Mhi)     (8)
 MathType@MTEF@5@5@+=feaafiart1ev1aaatCvAUfKttLearuWrP9MDH5MBPbIqV92AaeXatLxBI9gBaebbnrfifHhDYfgasaacH8akY=wiFfYdH8Gipec8Eeeu0xXdbba9frFj0=OqFfea0dXdd9vqai=hGuQ8kuc9pgc9s8qqaq=dirpe0xb9q8qiLsFr0=vr0=vr0dc8meaabaqaciaacaGaaeqabaqabeGadaaakeaacqWGWbaCcqGGOaakcqWGebarcqGG8baFcqWGnbqtdaWgaaWcbaGaemiAaGgabeaakiabcMcaPiabg2da9maarafabaGaemiCaaNaeiikaGIaemiraqKaeiiFaWNaemyta00aaSbaaSqaaiabdIgaOjabdMgaPbqabaGccqGGPaqkaSqaaiabdMgaPbqab0Gaey4dIunakiaaxMaacaWLjaWaaeWaaeaacqaI4aaoaiaawIcacaGLPaaaaaa@470B@

Assuming that all of the models are equally likely *a priori*, the search for the DBN with maximum posterior probability is equivalent to searching the network with maximum marginal likelihood. The use of Gaussian distributions and models that are linear in the parameters makes the computation very efficient because the marginal likelihood can be calculated in closed form. Here, we show in details the calculation of the factor *p*(*D*|*M*_*hi*_).

If we have measurements at *T *time points (so that we observe *n *= *T *- 1 transitions), the likelihood function for each variable *i *is given by

*p*(*D*_*i*_|*θ*_*hi*_) = (*τ*_*i*_/(2*π*))^*n*/2^*exp*[-*τ*_*i*_(*y*_*i *_- *X*_*i*_*β*_*i*_)^*T *^(*y*_*i *_- *X*_*i*_*β*_*i*_)/2]     (9)

where *y*_*i *_= (*y*_*i*2_,...,*y*_*iT*_)^*T *^is the *n *× 1 vector of observations, *β*_*i *_= (*β*_*i*0_, *β*_*i*1_,...,*β*_*ip*(*i*)_)^*T *^is the vector of regression parameters, *X*_*i *_is the *n *× (*p*(*i*) + 1) matrix of regression coefficients. For example, the row *t *is (1, *y*_*i*1*t*_, *y*_*i*2*t*_,...,*y*_*ip*(*i*)*t*_) when the model in Equation (4) is used, and it is (1, *tanh*(*αy*_*i*1*t*_), *tanh*(*αy*_*i*2*t*_),...,*tanh*(*αy*_*ip*(*i*)*t*_)) when the nonlinear model in Equation (5) is used.

We use conjugate prior distributions for the parameters *θ*_*hi*_, that consist of the precision *τ*_*i *_and the vector of regression parameters *β*_*i *_[6]. Therefore, we use a Gamma distribution as prior for *τ*_*i*_:

τi∼Gamma(αi1,αi2)p(τi)=1αi2αi1Γ(αi1)τiαi1−1e−τi/αi2     (10)
 MathType@MTEF@5@5@+=feaafiart1ev1aaatCvAUfKttLearuWrP9MDH5MBPbIqV92AaeXatLxBI9gBaebbnrfifHhDYfgasaacH8akY=wiFfYdH8Gipec8Eeeu0xXdbba9frFj0=OqFfea0dXdd9vqai=hGuQ8kuc9pgc9s8qqaq=dirpe0xb9q8qiLsFr0=vr0=vr0dc8meaabaqaciaacaGaaeqabaqabeGadaaakeaafaqabeqacaaabaacciGae8hXdq3aaSbaaSqaaiabdMgaPbqabaGccqWI8iIocqWGhbWrcqWGHbqycqWGTbqBcqWGTbqBcqWGHbqycqGGOaakcqWFXoqydaWgaaWcbaGaemyAaKMaeGymaedabeaakiabcYcaSiab=f7aHnaaBaaaleaacqWGPbqAcqaIYaGmaeqaaOGaeiykaKcabaGaemiCaaNaeiikaGIae8hXdq3aaSbaaSqaaiabdMgaPbqabaGccqGGPaqkcqGH9aqpdaWcaaqaaiabigdaXaqaaiab=f7aHnaaDaaaleaacqWGPbqAcqaIYaGmaeaacqWFXoqydaWgaaadbaGaemyAaKMaeGymaedabeaaaaGccqqHtoWrcqGGOaakcqWFXoqydaWgaaWcbaGaemyAaKMaeGymaedabeaakiabcMcaPaaaaaGae8hXdq3aa0baaSqaaiabdMgaPbqaaiab=f7aHnaaBaaameaacqWGPbqAcqaIXaqmaeqaaSGaeyOeI0IaeGymaedaaOGaemyzau2aaWbaaSqabeaacqGHsislcqWFepaDdaWgaaadbaGaemyAaKgabeaaliabc+caViab=f7aHnaaBaaameaacqWGPbqAcqaIYaGmaeqaaaaakiaaxMaacaWLjaWaaeWaaeaacqaIXaqmcqaIWaamaiaawIcacaGLPaaaaaa@730E@

where

αi1=νi02,αi2=2νi0σi02
 MathType@MTEF@5@5@+=feaafiart1ev1aaatCvAUfKttLearuWrP9MDH5MBPbIqV92AaeXatLxBI9gBaebbnrfifHhDYfgasaacH8akY=wiFfYdH8Gipec8Eeeu0xXdbba9frFj0=OqFfea0dXdd9vqai=hGuQ8kuc9pgc9s8qqaq=dirpe0xb9q8qiLsFr0=vr0=vr0dc8meaabaqaciaacaGaaeqabaqabeGadaaakeaafaqabeqacaaabaacciGae8xSde2aaSbaaSqaaiabdMgaPjabigdaXaqabaGccqGH9aqpdaWcaaqaaiab=17aUnaaBaaaleaacqWGPbqAcqaIWaamaeqaaaGcbaGaeGOmaidaaiabcYcaSaqaaiab=f7aHnaaBaaaleaacqWGPbqAcqaIYaGmaeqaaOGaeyypa0ZaaSaaaeaacqaIYaGmaeaacqWF9oGBdaWgaaWcbaGaemyAaKMaeGimaadabeaakiab=n8aZnaaDaaaleaacqWGPbqAcqaIWaamaeaacqaIYaGmaaaaaaaaaaa@4777@

Conditionally on *τ*_*i*_, the prior density of the parameter vector *β*_*i *_is assumed to be multivariate Gaussian:

βi|τi∼N(βi0∗,(τiRi0)−1)     (11)
 MathType@MTEF@5@5@+=feaafiart1ev1aaatCvAUfKttLearuWrP9MDH5MBPbIqV92AaeXatLxBI9gBaebbnrfifHhDYfgasaacH8akY=wiFfYdH8Gipec8Eeeu0xXdbba9frFj0=OqFfea0dXdd9vqai=hGuQ8kuc9pgc9s8qqaq=dirpe0xb9q8qiLsFr0=vr0=vr0dc8meaabaqaciaacaGaaeqabaqabeGadaaakeaaiiGacqWFYoGydaWgaaWcbaGaemyAaKgabeaakiabcYha8jab=r8a0naaBaaaleaacqWGPbqAaeqaaOGaeSipIOJaemOta4KaeiikaGIae8NSdi2aa0baaSqaaiabdMgaPjabicdaWaqaaiabgEHiQaaakiabcYcaSiabcIcaOiab=r8a0naaBaaaleaacqWGPbqAaeqaaOGaemOuai1aaSbaaSqaaiabdMgaPjabicdaWaqabaGccqGGPaqkdaahaaWcbeqaaiabgkHiTiabigdaXaaakiabcMcaPiaaxMaacaWLjaWaaeWaaeaacqaIXaqmcqaIXaqmaiaawIcacaGLPaaaaaa@4E11@

We set βi0∗
 MathType@MTEF@5@5@+=feaafiart1ev1aaatCvAUfKttLearuWrP9MDH5MBPbIqV92AaeXatLxBI9gBaebbnrfifHhDYfgasaacH8akY=wiFfYdH8Gipec8Eeeu0xXdbba9frFj0=OqFfea0dXdd9vqai=hGuQ8kuc9pgc9s8qqaq=dirpe0xb9q8qiLsFr0=vr0=vr0dc8meaabaqaciaacaGaaeqabaqabeGadaaakeaaiiGacqWFYoGydaqhaaWcbaGaemyAaKMaeGimaadabaGaey4fIOcaaaaa@31B9@ equal to zero and *R*_*i*0 _equal to the identity matrix. This choice represents the assumptions that all the variables are independent, and that, conditionally on *τ*_*i*_, the regression parameters are independent. Moreover, we chose *ν*_*i*0 _= 3 and σi02
 MathType@MTEF@5@5@+=feaafiart1ev1aaatCvAUfKttLearuWrP9MDH5MBPbIqV92AaeXatLxBI9gBaebbnrfifHhDYfgasaacH8akY=wiFfYdH8Gipec8Eeeu0xXdbba9frFj0=OqFfea0dXdd9vqai=hGuQ8kuc9pgc9s8qqaq=dirpe0xb9q8qiLsFr0=vr0=vr0dc8meaabaqaciaacaGaaeqabaqabeGadaaakeaaiiGacqWFdpWCdaqhaaWcbaGaemyAaKMaeGimaadabaGaeGOmaidaaaaa@31DE@ = 1 in order to have a large *a priori *variance σi2
 MathType@MTEF@5@5@+=feaafiart1ev1aaatCvAUfKttLearuWrP9MDH5MBPbIqV92AaeXatLxBI9gBaebbnrfifHhDYfgasaacH8akY=wiFfYdH8Gipec8Eeeu0xXdbba9frFj0=OqFfea0dXdd9vqai=hGuQ8kuc9pgc9s8qqaq=dirpe0xb9q8qiLsFr0=vr0=vr0dc8meaabaqaciaacaGaaeqabaqabeGadaaakeaaiiGacqWFdpWCdaqhaaWcbaGaemyAaKgabaGaeGOmaidaaaaa@30F0@.

With this prior specifications, it can be shown that the local marginal likelihood *p*(*D*|*M*_*hi*_) is given by:

p(D|Mhi)=1(2π)n/2(det⁡Ri0)1/2(det⁡Rin)1/2Γ(νin/2)Γ(νi0/2)(νi0σi02/2)νi0/2(νinσin2/2)νin/2     (12)
 MathType@MTEF@5@5@+=feaafiart1ev1aaatCvAUfKttLearuWrP9MDH5MBPbIqV92AaeXatLxBI9gBaebbnrfifHhDYfgasaacH8akY=wiFfYdH8Gipec8Eeeu0xXdbba9frFj0=OqFfea0dXdd9vqai=hGuQ8kuc9pgc9s8qqaq=dirpe0xb9q8qiLsFr0=vr0=vr0dc8meaabaqaciaacaGaaeqabaqabeGadaaakeaacqWGWbaCcqGGOaakcqWGebarcqGG8baFcqWGnbqtdaWgaaWcbaGaemiAaGMaemyAaKgabeaakiabcMcaPiabg2da9maalaaabaGaeGymaedabaGaeiikaGIaeGOmaidcciGae8hWdaNaeiykaKYaaWbaaSqabeaacqWGUbGBcqGGVaWlcqaIYaGmaaaaaOWaaSaaaeaacqGGOaakcyGGKbazcqGGLbqzcqGG0baDcqWGsbGudaWgaaWcbaGaemyAaKMaeGimaadabeaakiabcMcaPmaaCaaaleqabaGaeGymaeJaei4la8IaeGOmaidaaaGcbaGaeiikaGIagiizaqMaeiyzauMaeiiDaqNaemOuai1aaSbaaSqaaiabdMgaPjabd6gaUbqabaGccqGGPaqkdaahaaWcbeqaaiabigdaXiabc+caViabikdaYaaaaaGcdaWcaaqaaiabfo5ahjabcIcaOiab=17aUnaaBaaaleaacqWGPbqAcqWGUbGBaeqaaOGaei4la8IaeGOmaiJaeiykaKcabaGaeu4KdCKaeiikaGIae8xVd42aaSbaaSqaaiabdMgaPjabicdaWaqabaGccqGGVaWlcqaIYaGmcqGGPaqkaaWaaSaaaeaacqGGOaakcqWF9oGBdaWgaaWcbaGaemyAaKMaeGimaadabeaakiab=n8aZnaaDaaaleaacqWGPbqAcqaIWaamaeaacqaIYaGmaaGccqGGVaWlcqaIYaGmcqGGPaqkdaahaaWcbeqaaiab=17aUnaaBaaameaacqWGPbqAcqaIWaamaeqaaSGaei4la8IaeGOmaidaaaGcbaGaeiikaGIae8xVd42aaSbaaSqaaiabdMgaPjabd6gaUbqabaGccqWFdpWCdaqhaaWcbaGaemyAaKMaemOBa4gabaGaeGOmaidaaOGaei4la8IaeGOmaiJaeiykaKYaaWbaaSqabeaacqWF9oGBdaWgaaadbaGaemyAaKMaemOBa4gabeaaliabc+caViabikdaYaaaaaGccaWLjaGaaCzcamaabmaabaGaeGymaeJaeGOmaidacaGLOaGaayzkaaaaaa@9901@

where:

*α*_*i*1*n *_= *ν*_*i*0_/2 + *n*/2

Rin=Ri0+XiTXi
 MathType@MTEF@5@5@+=feaafiart1ev1aaatCvAUfKttLearuWrP9MDH5MBPbIqV92AaeXatLxBI9gBaebbnrfifHhDYfgasaacH8akY=wiFfYdH8Gipec8Eeeu0xXdbba9frFj0=OqFfea0dXdd9vqai=hGuQ8kuc9pgc9s8qqaq=dirpe0xb9q8qiLsFr0=vr0=vr0dc8meaabaqaciaacaGaaeqabaqabeGadaaakeaacqWGsbGudaWgaaWcbaGaemyAaKMaemOBa4gabeaakiabg2da9iabdkfasnaaBaaaleaacqWGPbqAcqaIWaamaeqaaOGaey4kaSIaemiwaG1aa0baaSqaaiabdMgaPbqaaiabdsfaubaakiabdIfaynaaBaaaleaacqWGPbqAaeqaaaaa@3D1F@

βin=Rin−1(Ri0βi0∗+XiTyi)
 MathType@MTEF@5@5@+=feaafiart1ev1aaatCvAUfKttLearuWrP9MDH5MBPbIqV92AaeXatLxBI9gBaebbnrfifHhDYfgasaacH8akY=wiFfYdH8Gipec8Eeeu0xXdbba9frFj0=OqFfea0dXdd9vqai=hGuQ8kuc9pgc9s8qqaq=dirpe0xb9q8qiLsFr0=vr0=vr0dc8meaabaqaciaacaGaaeqabaqabeGadaaakeaaiiGacqWFYoGydaWgaaWcbaGaemyAaKMaemOBa4gabeaakiabg2da9iabdkfasnaaDaaaleaacqWGPbqAcqWGUbGBaeaacqGHsislcqaIXaqmaaGccqGGOaakcqWGsbGudaWgaaWcbaGaemyAaKMaeGimaadabeaakiab=j7aInaaDaaaleaacqWGPbqAcqaIWaamaeaacqGHxiIkaaGccqGHRaWkcqWGybawdaqhaaWcbaGaemyAaKgabaGaemivaqfaaOGaemyEaK3aaSbaaSqaaiabdMgaPbqabaGccqGGPaqkaaa@4AA4@

1/αi2n=(−βinTRinβin+yiTyi+βi0∗TRi0βi0∗)/2+1/αi2
 MathType@MTEF@5@5@+=feaafiart1ev1aaatCvAUfKttLearuWrP9MDH5MBPbIqV92AaeXatLxBI9gBaebbnrfifHhDYfgasaacH8akY=wiFfYdH8Gipec8Eeeu0xXdbba9frFj0=OqFfea0dXdd9vqai=hGuQ8kuc9pgc9s8qqaq=dirpe0xb9q8qiLsFr0=vr0=vr0dc8meaabaqaciaacaGaaeqabaqabeGadaaakeaacqaIXaqmcqGGVaWliiGacqWFXoqydaWgaaWcbaGaemyAaKMaeGOmaiJaemOBa4gabeaakiabg2da9iabcIcaOiabgkHiTiab=j7aInaaDaaaleaacqWGPbqAcqWGUbGBaeaacqWGubavaaGccqWGsbGudaWgaaWcbaGaemyAaKMaemOBa4gabeaakiab=j7aInaaBaaaleaacqWGPbqAcqWGUbGBaeqaaOGaey4kaSIaemyEaK3aa0baaSqaaiabdMgaPbqaaiabdsfaubaakiabdMha5naaBaaaleaacqWGPbqAaeqaaOGaey4kaSIae8NSdi2aa0baaSqaaiabdMgaPjabicdaWaqaaiabgEHiQiabdsfaubaakiabdkfasnaaBaaaleaacqWGPbqAcqaIWaamaeqaaOGae8NSdi2aa0baaSqaaiabdMgaPjabicdaWaqaaiabgEHiQaaakiabcMcaPiabc+caViabikdaYiabgUcaRiabigdaXiabc+caViab=f7aHnaaBaaaleaacqWGPbqAcqaIYaGmaeqaaaaa@66D2@

*ν*_*in *_= *ν*_*i*0 _+ *n*

σin2=2/(νinαi2n)
 MathType@MTEF@5@5@+=feaafiart1ev1aaatCvAUfKttLearuWrP9MDH5MBPbIqV92AaeXatLxBI9gBaebbnrfifHhDYfgasaacH8akY=wiFfYdH8Gipec8Eeeu0xXdbba9frFj0=OqFfea0dXdd9vqai=hGuQ8kuc9pgc9s8qqaq=dirpe0xb9q8qiLsFr0=vr0=vr0dc8meaabaqaciaacaGaaeqabaqabeGadaaakeaaiiGacqWFdpWCdaqhaaWcbaGaemyAaKMaemOBa4gabaGaeGOmaidaaOGaeyypa0JaeGOmaiJaei4la8IaeiikaGIae8xVd42aaSbaaSqaaiabdMgaPjabd6gaUbqabaGccqWFXoqydaWgaaWcbaGaemyAaKMaeGOmaiJaemOBa4gabeaakiabcMcaPaaa@411A@

As parameter estimates, we consider their posterior expectations:

E(τi|yi)=αi1nαi2n=1/σin2
 MathType@MTEF@5@5@+=feaafiart1ev1aaatCvAUfKttLearuWrP9MDH5MBPbIqV92AaeXatLxBI9gBaebbnrfifHhDYfgasaacH8akY=wiFfYdH8Gipec8Eeeu0xXdbba9frFj0=OqFfea0dXdd9vqai=hGuQ8kuc9pgc9s8qqaq=dirpe0xb9q8qiLsFr0=vr0=vr0dc8meaabaqaciaacaGaaeqabaqabeGadaaakeaacqWGfbqrcqGGOaakiiGacqWFepaDdaWgaaWcbaGaemyAaKgabeaakiabcYha8jabdMha5naaBaaaleaacqWGPbqAaeqaaOGaeiykaKIaeyypa0Jae8xSde2aaSbaaSqaaiabdMgaPjabigdaXiabd6gaUbqabaGccqWFXoqydaWgaaWcbaGaemyAaKMaeGOmaiJaemOBa4gabeaakiabg2da9iabigdaXiabc+caViab=n8aZnaaDaaaleaacqWGPbqAcqWGUbGBaeaacqaIYaGmaaaaaa@4BDB@

*E*(*β*_*i*_|*y*_*i*_) = *β*_*in*_

E(σi2|yi)=νinσin2/(νin−2)
 MathType@MTEF@5@5@+=feaafiart1ev1aaatCvAUfKttLearuWrP9MDH5MBPbIqV92AaeXatLxBI9gBaebbnrfifHhDYfgasaacH8akY=wiFfYdH8Gipec8Eeeu0xXdbba9frFj0=OqFfea0dXdd9vqai=hGuQ8kuc9pgc9s8qqaq=dirpe0xb9q8qiLsFr0=vr0=vr0dc8meaabaqaciaacaGaaeqabaqabeGadaaakeaacqWGfbqrcqGGOaakiiGacqWFdpWCdaqhaaWcbaGaemyAaKgabaGaeGOmaidaaOGaeiiFaWNaemyEaK3aaSbaaSqaaiabdMgaPbqabaGccqGGPaqkcqGH9aqpcqWF9oGBdaWgaaWcbaGaemyAaKMaemOBa4gabeaakiab=n8aZnaaDaaaleaacqWGPbqAcqWGUbGBaeaacqaIYaGmaaGccqGGVaWlcqGGOaakcqWF9oGBdaWgaaWcbaGaemyAaKMaemOBa4gabeaakiabgkHiTiabikdaYiabcMcaPaaa@4CC1@

#### Search strategy

By the likelihood modularity described with the factorization in Equation (8), it is possible to learn the network structure by searching for the local regression models with maximum marginal likelihood. In the context of DBNs, time imposes a natural restriction on the set of candidate parents for each variable, because the parents are constrained to be the variables at the previous time point. However, even with this restriction the space of possible parent sets is exponential in the number of candidate parents. To make the search feasible, we adapted the greedy search strategy originally implemented in the K2 algorithm [26]. The algorithm evaluates models of increasing complexity as long as there is a gain in the marginal likelihood and stops when adding any extra parent to the current best model does not increase the marginal likelihood.

**Figure 10:**
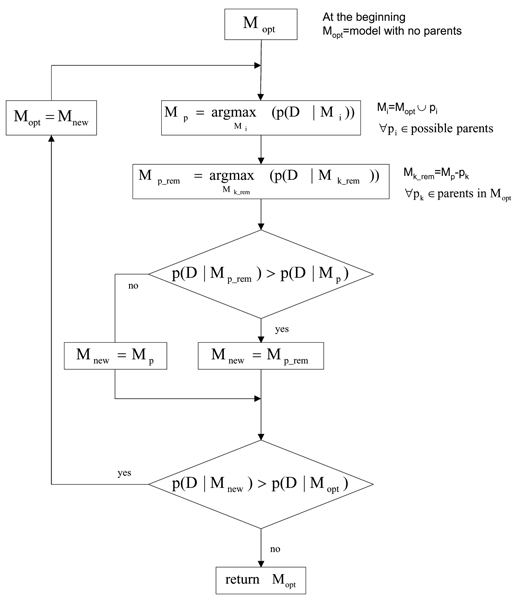
**Search strategy**. Schematic illustration of the strategy used to look for the parent set of each variable (see Methods).

In order to reduce the risk of finding suboptimal models, we implemented a stepwise search: at each step, the old marginal likelihood is not only compared with the marginal likelihood of the model in which the parent that increases the likelihood most is added to the old parent set, but also with the marginal likelihood values of the models in which this new parent is added to the old parent set with one of the old parents removed. The search strategy is schematically illustrated in Figure 10.

## Abbreviations

DBN: dynamic Bayesian network

BN: Bayesian network

RMSE: root mean squared error

R: recall

P: precision

TP: true positives

FP: false positives

FN: false negatives

F: F-measure

TN: true negatives

CV: coefficient of variation

## Competing interests

The authors declare that they have no competing interests.

## Authors' contributions

FF was responsible for the study: she conceived it together with RB, implemented the system, carried out the analyses, and wrote the manuscript. PS provided methodological support for all of the aspects related to the linear Gaussian network algorithm, and reviewed the paper. MFR supervised methodological and implementation aspects. RB contributed to the conception of the simulation study and the evaluation of the results, suggested the use of nonlinear Gaussian models, reviewed the paper, and supervised the work. All of the authors have read and approved the final manuscript.

## Figures and Tables

**Table 1:** Recall, precision and F-measures obtained when analyzing the data generated by the simulator developed by Yu et al. [15].

**Model**	**Data Points**	**Recall**	**Precision**	**F-measure**
Linear	100	0.9	0.58	0.7
Nonlinear (*α *= 1)	100	0.91	0.42	0.57
Linear	300	0.98	0.72	0.83
Nonlinear (*α *= 1)	300	0.98	0.57	0.72

For each number of time points (100 or 300), the values are averaged over 10 datasets, each corresponding to a different network structure.
